# Radiation pneumonia predictive model for radiotherapy in esophageal carcinoma patients

**DOI:** 10.1186/s12885-023-11499-6

**Published:** 2023-10-17

**Authors:** Liming Sheng, Lei Zhuang, Jing Yang, Danhong Zhang, Ying Chen, Jie Zhang, Shengye Wang, Guoping Shan, Xianghui Du, Xue Bai

**Affiliations:** 1 Zhejiang Key Laboratory of Radiation Oncology, Zhejiang Cancer Hospital, Hangzhou, Zhejiang 310022 China; 2 The Second Clinical Medical College, Zhejiang Chinese Medical University, Hangzhou, 310053 China

**Keywords:** Radiation pneumonia, Predictive model, Esophageal carcinoma, Radiotherapy, Machine learning, Deep learning

## Abstract

**Background:**

The machine learning models with dose factors and the deep learning models with dose distribution matrix have been used to building lung toxics models for radiotherapy and achieve promising results. However, few studies have integrated clinical features into deep learning models. This study aimed to explore the role of three-dimension dose distribution and clinical features in predicting radiation pneumonitis (RP) in esophageal cancer patients after radiotherapy and designed a new hybrid deep learning network to predict the incidence of RP.

**Methods:**

A total of 105 esophageal cancer patients previously treated with radiotherapy were enrolled in this study. The three-dimension (3D) dose distributions within the lung were extracted from the treatment planning system, converted into 3D matrixes and used as inputs to predict RP with ResNet. In total, 15 clinical factors were normalized and converted into one-dimension (1D) matrixes. A new prediction model (HybridNet) was then built based on a hybrid deep learning network, which combined 3D ResNet18 and 1D convolution layers. Machine learning-based prediction models, which use the traditional dosiomic factors with and without the clinical factors as inputs, were also constructed and their predictive performance compared with that of HybridNet using tenfold cross validation. Accuracy and area under the receiver operator characteristic curve (AUC) were used to evaluate the model effect. DeLong test was used to compare the prediction results of the models.

**Results:**

The deep learning-based model achieved superior prediction results compared with machine learning-based models. ResNet performed best in the group that only considered dose factors (accuracy, 0.78 ± 0.05; AUC, 0.82 ± 0.25), whereas HybridNet performed best in the group that considered both dose factors and clinical factors (accuracy, 0.85 ± 0.13; AUC, 0.91 ± 0.09). HybridNet had higher accuracy than that of Resnet (*p* = 0.009).

**Conclusion:**

Based on prediction results, the proposed HybridNet model could predict RP in esophageal cancer patients after radiotherapy with significantly higher accuracy, suggesting its potential as a useful tool for clinical decision-making. This study demonstrated that the information in dose distribution is worth further exploration, and combining multiple types of features contributes to predict radiotherapy response.

**Supplementary Information:**

The online version contains supplementary material available at 10.1186/s12885-023-11499-6.

## Background

Radiotherapy is the mainstay treatment for esophageal carcinoma, and the radiation pneumonia (RP) is one of the most serious complications of thoracic radiotherapy [1, 2]. RP affects the normal progress of radiotherapy and causes a decline in patients’ quality of life. Previous studies suggest that radiation dose is a key risk factor for the development of radiotherapy-induced lung injury, including mean lung dose and dose-volume histogram parameters such as lung V_5_, V_10_, V_20_, and V_30_ [3–8]. Advances in computer science have enabled researchers to describe the radiation effects more explicitly, using three-dimensional dose distribution features [9–11]. This has led to the development of dosiomics, a method derived from radiomics, which uses mathematical transformations to extract the spatial characteristics of dose distribution to predict radiation response [12]. Liang et al. used the dosiomic method to extract dosiomic features from the lung dose distribution in non-small cell lung cancer patients undergoing radiotherapy, and demonstrated that using dosiomics characteristics can improve the accuracy of the prediction model for RP in this population [9]. In their follow-up study, they extended the model to a dual-omics model that combined dosiomic characteristics and ventilation image features and implemented it using deep learning technique to achieve a better predictive performance [13].

Despite the promising results from using dosiomic parameters and deep learning models explore the value of dose distribution in predicting the risk of RP, few studies have integrated clinical features into these models. Several studies have suggested that in addition to the absorbed dose, other factors, such as basic diseases, biological markers, gene polymorphism, lung function and mode of treatment can also influence the incidence of RP. At present, it is generally considered that the occurrence of RP is a complex process that is influenced by both clinical factors and dosimetry factors [14–19]. However, clinical factors have not been combined with dosiomic features in deep learning RP prediction models. In addition, most dosiomic and deep learning studies have focused on lung cancer, with relatively few studies examining esophageal cancer [13].

In this study, we designed a hybrid deep learning model, which combined dose distribution and clinical characteristics to predict RP. The hybrid model outperformed dosiomics-based and machine learning-based models in predictive accuracy for RP in patients with locally advanced esophageal cancer receiving radical radiotherapy and chemotherapy.

## Patients and methods

### Patients

Patients with esophageal cancer who underwent radical radiotherapy in Zhejiang Cancer Hospital from January 2020 to August 2021 were included in this study. The inclusion criteria were as followed: Newly histologically or cytologically confirmed esophageal squamous cell carcinoma; at the least two cycles of immunotherapy combined with chemotherapy before radiotherapy; without surgery; a total radiotherapy dose of more than 50 Gy; completion of chest radiotherapy. The exclusion criteria included active coexisting cancer; receiving surgery before or after chemoradiotherapy or a total radiotherapy dose of less than 50 Gy. In total, 105 patients were included in this study. All patients received 6-MV X-ray external radiotherapy, with a prescription plan target volume (PTV) dose of 50–61.6 Gy. Among them, 98 cases were treated with linacs and 7 cases with tomography. Of the patients treated with linacs, 95 were planned in the Raystaion Ver9.0 planning system (RaySearch Laboratories AB, Sweden) and 3 in the Eclipse Ver15.0 planning system (Varian, USA). Tomotherapy planning was completed using HiArt TomoTherapy Planning system (Accuray Inc., USA). CT images of patients were obtained using Brilliance Big Bore CT (Philips Medical Systems, Cleveland, USA) and a slice thickness of 5 mm. All plans were normalized at 95% of the prescribed dose of PTV. The lung tissue was first delineated automatically using the threshold segmentation method and then corrected by the physicians. Patients' cough, sputum and respiration were observed after radiotherapy, and chest CT scan was performed if necessary. RP was assessed using the National Cancer Institute's Common Terminology Criteria for Adverse Events (CTCAE) Version 5.0 [20]. The endpoint was RP (grade ≥ 2) occurring within 6 months after initiating radiotherapy. All toxicities occurring at 1, 3, and 6 months were included.

### Data preprocessing

**Fig. 1:**
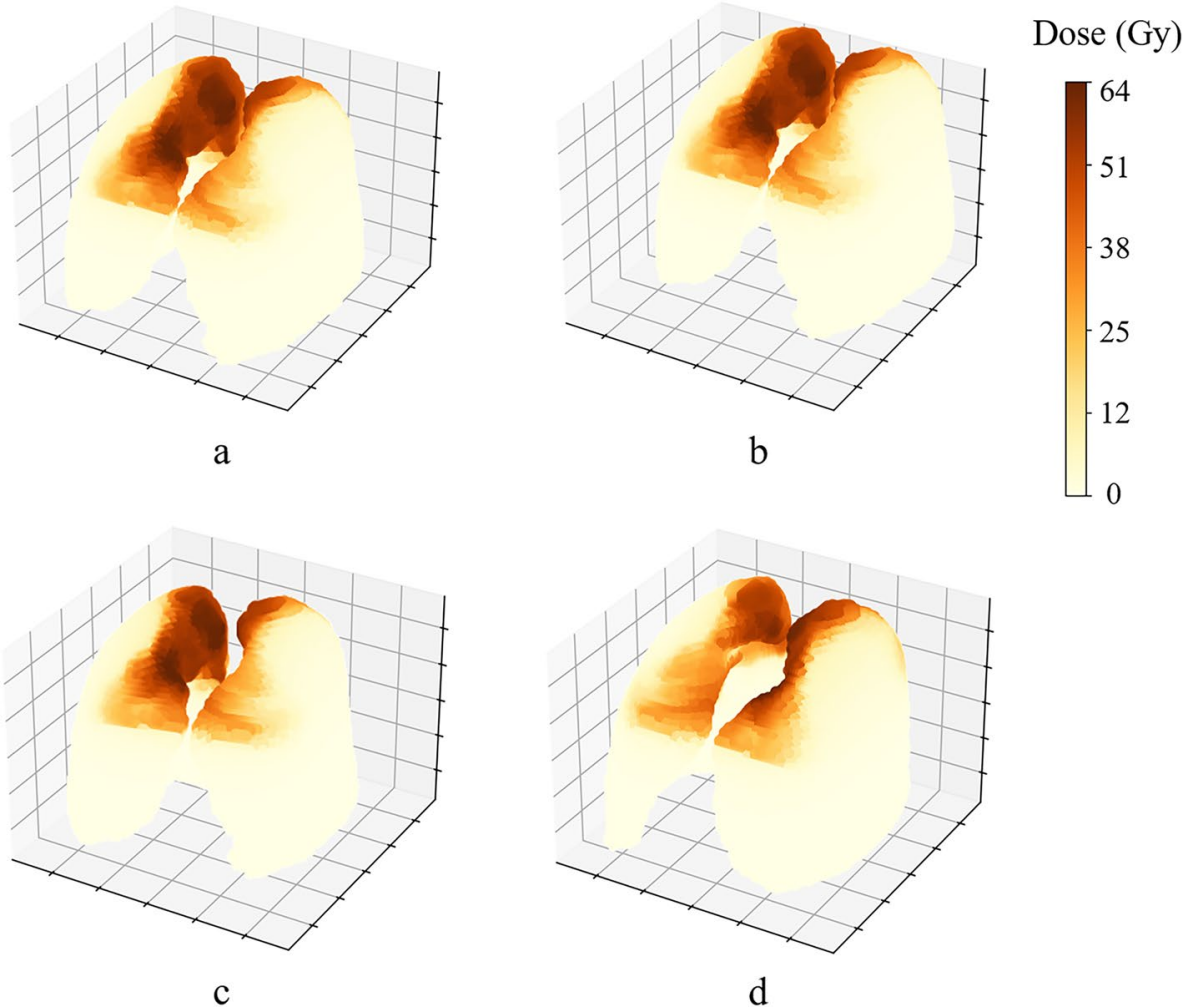
An example of lung 3D dose matrix (**a**) and its deformation after using the image augmentation techniques. Subfigure **b**, **c**, and **d** represent shift, rotate and flip, respectively

In this study, clinical factors and radiotherapy dose distributions were used to predict RP. All data was anonymized before analysis. There were 15 clinical features, including PTV volume, chemotherapy regimen, immunotherapy drugs, age, gender, smoking status, T stage, N stage, tumor location, tumor length, immunotherapy period, concurrent chemotherapy, consolidative immunotheray, consolidative chemotherapy, and the interval between immunotherapy and radiotherapy. Patient demographics and details of the features are shown in Table 1. The dose distributions inside the lung were derived from DICOM data in Gy, resampled to a pixel size of 2.52.52.5 mm^3^, and then converted to 3-dimension (3D) matrixes. The elements in the area outside the lung contour were signed 0. One of the 3D dose matrixes is shown in Fig. 1(a).

**Table 1:** Summary of patient data

Continuous variables	Median (Range)	
PTV Volume (cc)	410.38 (1021.67–98.15)	
Age (years)	69 (44–79)	
tumor length (cm)	6.0 (1.0–14.0)	
interval between immunotherapy and radiotherapy (days)	36 (1–98)	
Ordinal Categorical Variables		
Cycles of ICI	3 (1–4)	
Cycles of chemotherapy	0 (0–5)	
Cycles of consolidative ICI	0 (0–3)	
T stage	1	
	2	
	3	
	4	
N stage	1	
	2	
	3	
	4	
Nominal categorical variables	Occurrence	
chemotherapy regimen	albumin-bound paclitaxel	90
	paclitaxel	15
immunotherapy drugs	camrelizumab	65
	pembrolizumab	13
	durvalumab	7
	toripalimab	1
	Sintilimab	11
	Nivolumab	2
	Tislelizumab	6
gender	Male	99
	female	6
smoking status	Yes	80
	No	25
tumor location	Cervical	4
	Upper thoracic	13
	Middle thoracic	49
	Lower thoracic	37
	Whole esophagus	2
concurrent chemotherapy	Yes	45
	No	60
Outcome	Occurrence	
RP	Yes	23
	No	82

### RP prediction model

**Fig. 2:**
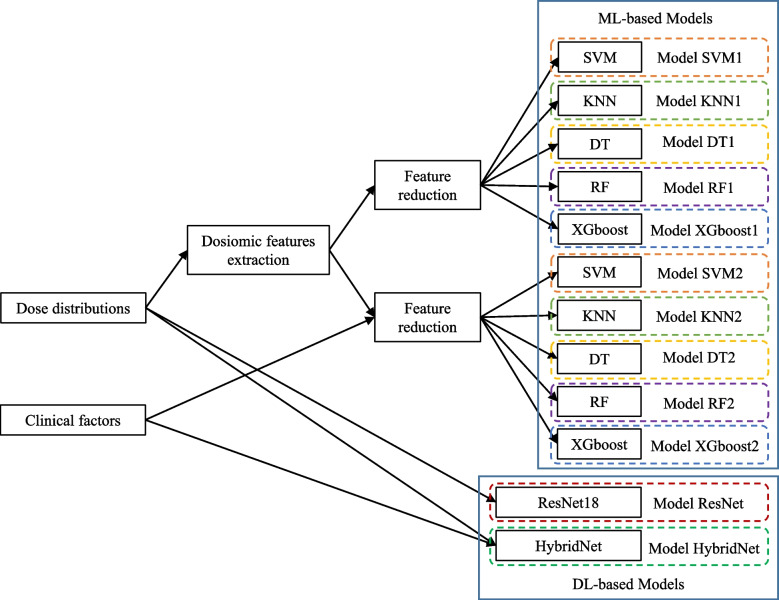
Experiment design. Two types of models: machine learning (ML)-based and deep learning (DL)-based models were tested

The purpose of this study was to design and evaluate a hybrid deep learning model that could directly integrate lung dose matrix and clinical features as inputs and generate the probability of RP as the output. To evaluate the predictive ability of the proposed model, we compared the machine learning models that extracted dosiomic features first with the deep learning model that only considered the dose matrix without clinical features. Therefore, we built two kinds of models to predict RP: a machine learning-based model and 3D deep learning-based model that used dosiomic features and 3D lung dose distribution matrix with and without clinical features as input data, respectively. The design of this experiment is shown in Fig. 2.

### ML based classifier

#### Dosiomic features extract

In total, 128 dosiomic features were extracted from the lung dose distribution. A dosiomic feature extraction method similar to the radiomic features extraction method was completed using python-based pyradiomics Ver3.0 library function [21], which complies with recommendations for standardizing feature extraction by the Image Biomarker Standardisation Initiative [22]. Shape features were computed from the region with a dose larger than 5 Gy, 20 Gy and 30 Gy inside the lung and each region had 14 features. In total, there were 18 first-order features, 22 Gy co-occurrence matrix (GLCM) features, 16 Gy run matrix (GLRLM) features, 16 Gy area size matrix (GLSZM) features and 14 Gy correlation matrix (GLDM) features. The bin width in feature extraction was set to 1. Since the numerical unit of the dose distribution matrix was Gy, a bin width of 1 meant that the matrix was discretized with 1 Gy. Feature extraction calculation was performed using previously described formulas [21]. All the dosiomic features were listed in Additional file 1.

#### Feature selection and reduction

Features were selected using univariate logistic regression analysis and used to investigate features significantly associated with the RP. A *p* value < 0.05 was considered significant. All redundancy features were excluded. Nominal categorical variables shown in Table 1 were transformed into dummy variables. Before analysis, all the features were standardized to have a mean of 0value and a standard deviation of 1 with the stander scale. To avoid model over-fitting due to too many clinical and dosiomic features, principle component analysis (PCA) was used to further reduce the number of features after univariate logistic regression analysis. The PCA reconstruction threshold of 0.9 was used, which meant that the sum of the eigenvalues of the reduced dimension variables accounts for 90% of the total variance in the original data.

#### Prediction models

Five most commonly used ML-based binary classifiers were trained for RP prediction. These included the support vector machine (SVM), k-nearest neighbors (KNN), decision tree (DT), random forest (RF) and eXtreme Gradient Boosting (XGboost). The hyper-parameters of each classifier were repeatedly adjusted by grid-search method to obtain the best prediction value. For SVM classifier, the kernel format was linear, and the C was 0.8. For KNN classifier, the number of neighbors used was 30. For XGboost classifier, the max depth was 10, the learning rate was 0.1, and the number of estimators was 200. Except as mentioned above, all hyper-parameters used the default values of Scikit-learn package (V0.24.2) in python language [23].

### Deep learning based classifier

#### ResNet

The RseNet18 architecture, which has been successfully applied in image classification, was used to predict RP. The elemental structure of ResNet18 was the Conv-BN-ReLU. The convolution and batch normalization were performed in the elemental structure first followed by rectified linear unit (ReLU) activation. There were two kinds of blocks in the network. The first block was composed of four sequential Conv-BN-ReLUs with the skip connection, whereas the second block comprised five Conv-BN-ReLU structures with 4 sequential arrangements and one skip connection. Overall, the RseNet18 network contained 1 Conv-BN-ReLU, 1 block 1, 3 block 2 and 1 fully connection layer [24]. In this study, the 3D lung dose matrixes were used as inputs and the RP results were the output.

#### HybridNet

**Fig. 3:**
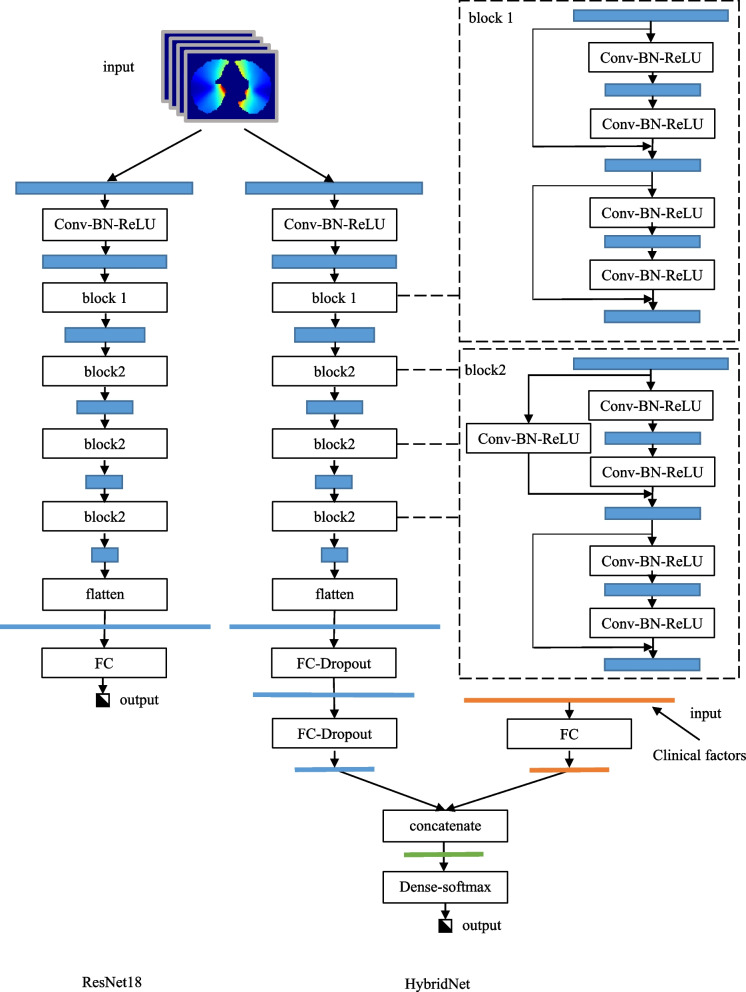
The RseNet18 and HybridNet architecture

In this study, a hybrid net was designed based on Resnet18 to combine clinical factors with dose matrixes. The last fully connection layer in Resnet18 was alternated by adding two fully connection layers with dropout rate 0.5. The dropout was used to mitigate overfitting, which was likely since the number of cases was far less than the features extracted. This is a common issue in deep learning. In the HybridNet, a fully connection layer parallel with the ResNet18 was designed to process with the clinical factors. A concatenate layer was used to combine the dose features and the clinical factors. The predict results were outputted through a dense-softmax layer at the end of the net. The ResNet18 and HybridNet architecture is shown in Fig. 3. Both ResNet and HybridNet used the same optimized parameters. The RP results were converted to one-hot coding and the cross entropy loss function was used to guide the Adam optimizer. The initial learning rate was 0.1 and adjusted by a factor of 0.01 every five epochs if the loss function remained constant. The limit of learning rate was 0.510^–5^. The batch size was 2, and the maximum number of epochs was 500. If the loss difference in two adjunct epochs was less than 10^–4^ and lasted for 10 epochs, the training would stop early. The deep learning network was built using the Keras library (V.2.7.0) in python language [25].

## Model training and validation

The tenfold cross-validation was used to evaluate the prediction performance of the model. The dataset was randomly divided into 10 equal parts and stratified sampling was used to ensure an equal proportion of negative and positive cases in each part. Among them, 9 were used as training and validation data and 1 as test data for the models. The hyper-parameters of each classifier were tuned and validated on the validation subset. To avoid over fitting, during the training phase of the ResNet and the HybridNet, the random rotation range and random width and height shift were set to ± 20°and ± 5 cm, and random left–right flipping was adopted for data augmentation. The example of the image augmentation performed on a 3D lung dose matrix were shown in Fig. 1(b, c, and d). The prediction performance of the model was quantitatively evaluated using two metrics: the average value of the accuracy and the area under the working characteristic curve (AUC) of 10 models. The accuracy was defined as the frequency with which prediction result matched the follow-up result, and was calculated by:

$$\mathrm{accuracy}=\frac{TP+TN}{TP+TN+FP+FN}$$

where TP, TN, FP and FN meant the number of true positive, true negative, false positive, and false negative, respectively. The AUC was calculated by:

$$\mathrm{AUC}=\frac{\sum I({P}_{positive},{P}_{negtive})}{M\times N}$$

where M and N was the number of positive and negative, respectively. $I\left({P}_{positive},{P}_{negtive}\right)$ was calculated by:

$$I\left({P}_{positive},{P}_{negtive}\right)=\left\{\begin{array}{c}1, {P}_{positive}>{P}_{negtive}\\ 0.5, {P}_{positive}={P}_{negtive}\\ 0, {P}_{positive}<{P}_{negtive}\end{array}\right.$$

where ${P}_{positive}$ and ${P}_{negtive}$ was the predicted probability of positive and negative, respectively. The ML classifier building and the cross validation were implemented using the Scikit-learn package (V0.24.2) in python language [23].

### Statistical analysis

To evaluate the differences between the models constructed using different features, the data were randomly divided into training set and test set at 1:1 ratio. The Delong test was then performed to evaluate the significant differences between the models using an in-house program in python language [26]. A *p* value < 0.05 was considered significant.

## Results

Among the 105 patients enrolled in this study, 23 (21.9%) developed RP of grade 2 or above after radiotherapy, including 16, 6, and 1 cases with grade 2, 3, and 5 RP, respectively.

The results of univariate logistic regression analysis are shown in Table 2. Out of a total of 143 features used, 83 were significantly correlated with the incidence of RP. In the 15 clinical features, the cycles of immune checkpoint inhibitor (ICI) were positively correlated with RP incidence. In the 128 dosiomic features, 82 features were significantly different between the RP group and the non RP group. The detail of analysis results was listed in Additional file 1. After applying PCA to the features, the feature dimensions reduced to 6.

**Table 2:** The results of univariate logistic regression analysis

		total features	correlated features
Clinical features		15	1
Dosiomic features	500cGyROI shape features	14	6
	2000cGyROI shape features	14	6
	3000cGyROI shape features	14	8
	First order features	18	16
	GLCM features	22	19
	GLRLM features	16	12
	GLSZM features	16	3
	GLDM features	14	12
Total		143	83

**Fig. 4:**
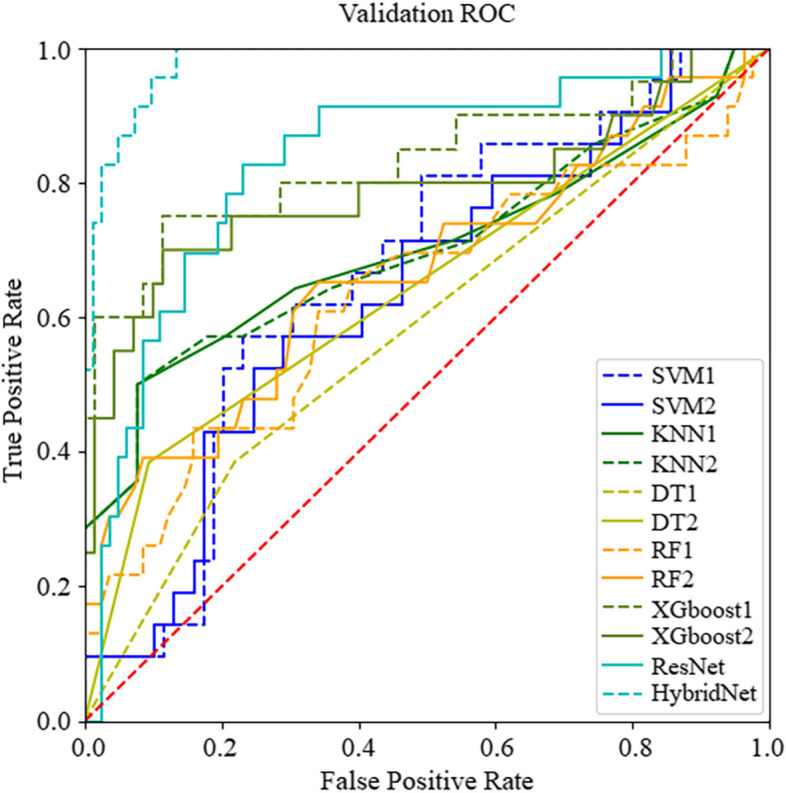
ROC curves of predictive models

Table 3 shows the accuracy and AUC of each model. The results of DeLong test between the models constructed with and without clinical features are shown in the last column. For the DT, XGboost and deep learning methods, the model incorporated clinical features significantly better than the model only using dosiomic or dose distribution features. ROC curves of the models are shown in Fig. 4.

**Table 3:** The accuracy and AUC of each model

	accuracy	AUC		accuracy	AUC	p
SVM1	0.68 ± 0.12	0.63 ± 0.20	SVM2	0.61 ± 0.12	0.68 ± 0.16	0.061
KNN1	0.78 ± 0.04	0.71 ± 0.18	KNN2	0.78 ± 0.04	0.68 ± 0.17	1.000
DT1	0.71 ± 0.14	0.61 ± 0.20	DT2	0.72 ± 0.12	0.62 ± 0.18	0.035
RF1	0.79 ± 0.09	0.60 ± 0.23	RF2	0.78 ± 0.07	0.68 ± 0.21	0.706
XGboost1	0.73 ± 0.12	0.51 ± 0.20	XGboost2	0.78 ± 0.09	0.61 ± 0.21	0.041
ResNet	0.78 ± 0.05	0.82 ± 0.25	HybridNet	0.85 ± 0.13	0.91 ± 0.09	0.009

## Discussion

As one of the most common complications of esophageal cancer radiotherapy, RP affects normal progress of radiotherapy and reduces the quality of life of patients. In the current study, a new prediction model HybridNet was proposed to predict the incidence rate of RP in esophageal cancer patients receiving radiotherapy. HybridNet used a deep convolution network to explore the dose distribution data of lungs and combined clinical features with dose information. The use of deep learning network helped improve RP prediction performance by mining as much information as possible from the dose distribution. We also designed experiments to study the value of integrating clinical factors into the RP prediction model. The results showed that combining clinical factors with dose information could effectively improve the prediction accuracy as long as appropriate models are selected, especially in deep learning-based models. The prediction model established in this study could accurately predict RP and identify esophageal carcinoma patients with a high risk of RP, and could serve as an effective tool in the planning evaluation stage to determine whether further improvements are needed to the current radiotherapy treatment plan.

Machine learning and deep learning are both cutting-edge mathematical methods in the medical field, and have achieved great success in fields such as automatic segmentation [27–30], radiotherapy dose prediction [31–34], automatic treatment planning [35–37], disease severity assessment [38], and prognosis evaluation [11, 39, 40]. Radiomics, the source of dosiomics, are also frontiers for analyzing medical images. A series of detailed and in-depth studies have been carried out on radiomics, such as the importance of repeatability and robustness of features [40, 41], and the progress from conventional radiomic features to tensor radiomic features [39, 42]. Dosiomics, on the other hand, has not yet been studied in such depth.

In this study, whether in the experimental group with or without clinical features, the deep learning-based classifier showed higher accuracy than ML-based classifier. In the ML model, we used the dosiomic method, which is one of the best feature extraction methods for mining information from radiotherapy dose distribution [9, 10, 12]. The dosiomic method could extract hundreds of features, including texture, shape and other information, far exceeding the number of traditional dose-volume histogram features. However, the dosiomic features calculation method is derived from radiomics, which is used to extract data from medical images for diagnosis, and the calculation method is not specifically optimized for predicting biological tissue reaction to radiation. In contrast, in the deep learning model, the feature extraction is not based on fixed mathematical formulas, but on the feedback adjustment of convolution filters according to the prediction results obtained during the training process. In addition, the model specifically mines information on biological effects in dose distribution. The deep learning network also outperforms traditional methods in predicting radiation-induced xerostomia [43] and provides a dual-omics prediction model with a better predictive value for RP [13].

Most previous deep learning-based prediction models only used dose distribution matrixes to predict biological effects, but these may not be optimal for predicting toxicity. To the best of our knowledge, this is the first study to incorporate clinical variables into deep learning network to predict the RP of esophageal cancer radiotherapy. As can be seen from Table 3, the effect of adding clinical characteristics on prediction performance varied between models. According to the results of the Delong test, after adding clinical features, the results of DT models improved, but to a less extent compared with XGboost and deep learning models. Methods using either dosiomics features or deep learning-extracted features aim to mine as much information as possible from dose distribution. In contrast, the description of clinical variables is so simple that important information may be ignored when the dose features is complex. Among the models tested in this study, XGboost and deep learning models were shown to better retain clinical features, providing valuable information for prediction.

The risk of over-fitting in deep learning models is common, especially when the amount of training set data is small. To overcome this limitation, the proposed HybridNet used data augmentation, regularization and dropout. In the training progress, the loss function declined for both the test set and validation set. The tenfold cross validation results showed that the accuracy and AUC were 0.87 ± 0.21 and 0.94 ± 0.15 for the validation set and 0.85 ± 0.13 and 0.91 ± 0.09 for the test set, respectively. No significant difference in performance was found between validation set and test set, suggesting that the methods effectively prevented the model from over-fitting.

The HybridNet architecture was based on the ResNet18. The ResNet uses residual blocks with skip connections to mitigate the problems caused by vanishing or exploding gradient in deep networks. Since ResNet18 is a classical and efficient classification network [24], it was chosen for predicting RP in this study. Optimization of the network structure in future work may further improve prediction performance.

Although the proposed HybridNet showed high predictive performance in RP, further research is still necessary to further improve its performance. One limitation of this study is that the effect of dose matrix parameters, such as dose grid resolution, dose calculation algorithm and the pixel spacing of dose cube, was not studied. As these factors have been shown to affect the reproducibility and stability of dosiomic features [44], it is likely that they could also affect the stability of deep learning models. Therefore, the effect of different input data parameters on the performance of HybridNet model needs further investigation. A second limitation is that other factors such as the ratio of T helper cells 17 and T regulatory cells, which strongly predict RP [45], were not considered in the HybridNet model. Therefore, our further work will attempt to include other relevant variables in the HybridNet. A third limitation is that the effect of respiratory exercise and setup error on dose distribution was not taken into account, and the substructures of the lung, such as bronchi, were not considered separately. A more accurate dose distribution calculation could further improve the predictive accuracy of the HybridNet model.

## Conclusion

This study designed a new prediction model HybridNet, which was based on deep learning network and combined clinical features with dose information for accurate RP prediction after radiotherapy. The HybridNet outperformed machine learning-based and dosiomics-based models and ResNet model using only dose matrixes as input. It achieved improved prediction of RP incidence, suggesting its potential as a useful tool for clinical decision-making.

### Supplementary Information

**Additional file 1.** The dosiomic features extracted from lung dose distribution and the results of univariate logistic regression analysis.

## Data Availability

All data generated or analyzed during this study are available from the corresponding author on reasonable request.

## Abbreviations

RP: Radiation pneumonitis;
AUC: Area under the receiver operator characteristic curve
1D: One dimension
3D: Three dimension
CTCAE: National Cancer Institute's Common Terminology Criteria for Adverse Events
GLCM: Gray co-occurrence matrix
GLRLM: Gray run matrix
GLSZM: Gray area size matrix
GLDM: Gray correlation matrix
PCA: Principle component analysis
SVM: Support vector machine
KNN: K-nearest neighbors
DT: Decision tree
RF: Random forest
XGboost: EXtreme Gradient Boosting
ReLU: Rectified linear unit
ML: Machine learning
DL: Deep learning
FC: Fully convolution
ICI: Immune checkpoint inhibitor
