# Supplementary material for: Radiation pneumonia predictive model for radiotherapy in esophageal carcinoma patients
Source: BMC Cancer. 2023 Oct 17;23:988. doi: 10.1186/s12885-023-11499-6 (PMC10580570; doi:10.1186/s12885-023-11499-6)
Supplement: Supplementary file 1 — Additional file 1. The dosiomic features extracted from lung dose distribution and the results of univariate logistic regression analysis. [file 12885_2023_11499_MOESM1_ESM.docx]

**Additional file 1**

The dosiomic features extracted from lung dose distribution and the results of univariate logistic regression analysis.

| feature types | features | Whether correlated with RP |
| --- | --- | --- |
| 500cGyROI shape features | Elongation |  |
|  | Flatness | 🗸 |
|  | LeastAxisLength | 🗸 |
|  | MajorAxisLength |  |
|  | Maximum2DDiameterColumn | 🗸 |
|  | Maximum2DDiameterRow |  |
|  | Maximum2DDiameterSlice |  |
|  | Maximum3DDiameter |  |
|  | MeshVolume | 🗸 |
|  | MinorAxisLength |  |
|  | Sphericity |  |
|  | SurfaceArea | 🗸 |
|  | SurfaceVolumeRatio |  |
|  | VoxelVolume | 🗸 |
| 2000cGyROI shape features | Elongation |  |
|  | Flatness |  |
|  | LeastAxisLength | 🗸 |
|  | MajorAxisLength |  |
|  | Maximum2DDiameterColumn |  |
|  | Maximum2DDiameterRow | 🗸 |
|  | Maximum2DDiameterSlice |  |
|  | Maximum3DDiameter |  |
|  | MeshVolume | 🗸 |
|  | MinorAxisLength | 🗸 |
|  | Sphericity |  |
|  | SurfaceArea | 🗸 |
|  | SurfaceVolumeRatio |  |
|  | VoxelVolume | 🗸 |
| 3000cGyROI shape features | Elongation |  |
|  | Flatness |  |
|  | LeastAxisLength |  |
|  | MajorAxisLength | 🗸 |
|  | Maximum2DDiameterColumn |  |
|  | Maximum2DDiameterRow | 🗸 |
|  | Maximum2DDiameterSlice | 🗸 |
|  | Maximum3DDiameter |  |
|  | MeshVolume | 🗸 |
|  | MinorAxisLength | 🗸 |
|  | Sphericity | 🗸 |
|  | SurfaceArea | 🗸 |
|  | SurfaceVolumeRatio |  |
|  | VoxelVolume | 🗸 |
| First order features | 10Percentile | 🗸 |
|  | 90Percentile | 🗸 |
|  | Energy | 🗸 |
|  | Entropy | 🗸 |
|  | InterquartileRange | 🗸 |
|  | Kurtosis |  |
|  | Maximum | 🗸 |
|  | MeanAbsoluteDeviation | 🗸 |
|  | Mean | 🗸 |
|  | Median | 🗸 |
|  | Minimum |  |
|  | Range | 🗸 |
|  | RobustMeanAbsoluteDeviation | 🗸 |
|  | RootMeanSquared | 🗸 |
|  | Skewness | 🗸 |
|  | TotalEnergy | 🗸 |
|  | Uniformity | 🗸 |
|  | Variance | 🗸 |
| GLCM features | Autocorrelation | 🗸 |
|  | JointAverage | 🗸 |
|  | ClusterProminence | 🗸 |
|  | ClusterShade | 🗸 |
|  | ClusterTendency | 🗸 |
|  | Contrast | 🗸 |
|  | Correlation |  |
|  | DifferenceAverage | 🗸 |
|  | DifferenceEntropy | 🗸 |
|  | DifferenceVariance | 🗸 |
|  | JointEnergy | 🗸 |
|  | JointEntropy | 🗸 |
|  | Imc1 | 🗸 |
|  | Imc2 |  |
|  | Idm | 🗸 |
|  | Idmn |  |
|  | Id | 🗸 |
|  | Idn | 🗸 |
|  | InverseVariance | 🗸 |
|  | MaximumProbability | 🗸 |
|  | SumEntropy | 🗸 |
|  | SumSquares | 🗸 |
| GLRLM features | GrayLevelNonUniformity |  |
|  | GrayLevelNonUniformityNormalized |  |
|  | GrayLevelVariance | 🗸 |
|  | HighGrayLevelRunEmphasis | 🗸 |
|  | LongRunEmphasis | 🗸 |
|  | LongRunHighGrayLevelEmphasis |  |
|  | LongRunLowGrayLevelEmphasis | 🗸 |
|  | LowGrayLevelRunEmphasis | 🗸 |
|  | RunEntropy |  |
|  | RunLengthNonUniformity | 🗸 |
|  | RunLengthNonUniformityNormalized | 🗸 |
|  | RunPercentage | 🗸 |
|  | RunVariance | 🗸 |
|  | ShortRunEmphasis | 🗸 |
|  | ShortRunHighGrayLevelEmphasis | 🗸 |
|  | ShortRunLowGrayLevelEmphasis | 🗸 |
| GLSZM features | GrayLevelNonUniformity | 🗸 |
|  | GrayLevelNonUniformityNormalized |  |
|  | GrayLevelVariance |  |
|  | HighGrayLevelZoneEmphasis |  |
|  | LargeAreaEmphasis |  |
|  | LargeAreaHighGrayLevelEmphasis |  |
|  | LargeAreaLowGrayLevelEmphasis |  |
|  | LowGrayLevelZoneEmphasis |  |
|  | SizeZoneNonUniformity | 🗸 |
|  | SizeZoneNonUniformityNormalized |  |
|  | SmallAreaEmphasis |  |
|  | SmallAreaHighGrayLevelEmphasis |  |
|  | SmallAreaLowGrayLevelEmphasis |  |
|  | ZoneEntropy |  |
|  | ZonePercentage | 🗸 |
|  | ZoneVariance |  |
| GLDM features | DependenceEntropy | 🗸 |
|  | DependenceNonUniformity | 🗸 |
|  | DependenceNonUniformityNormalized | 🗸 |
|  | DependenceVariance |  |
|  | GrayLevelNonUniformity | 🗸 |
|  | GrayLevelVariance | 🗸 |
|  | HighGrayLevelEmphasis | 🗸 |
|  | LargeDependenceEmphasis | 🗸 |
|  | LargeDependenceHighGrayLevelEmphasis |  |
|  | LargeDependenceLowGrayLevelEmphasis | 🗸 |
|  | LowGrayLevelEmphasis | 🗸 |
|  | SmallDependenceEmphasis | 🗸 |
|  | SmallDependenceHighGrayLevelEmphasis | 🗸 |
|  | SmallDependenceLowGrayLevelEmphasis | 🗸 |
